# Murine fecal microbiota transplantation lowers gastrointestinal pathogen loads and dampens pro-inflammatory immune responses in *Campylobacter jejuni* infected secondary abiotic mice

**DOI:** 10.1038/s41598-019-56442-7

**Published:** 2019-12-24

**Authors:** Markus M. Heimesaat, Katharina Mrazek, Stefan Bereswill

**Affiliations:** Institute of Microbiology, Infectious Diseases and Immunology, Gastrointestinal Microbiology Research Group, Charité - University Medicine Berlin, corporate member of Freie Universität Berlin, Humboldt-Universität zu Berlin, and Berlin Institute of Health, Berlin, Germany

**Keywords:** Bacterial infection, Bacteriology, Pathogens

## Abstract

Conventional mice are protected from *Campylobacter jejuni* infection by the murine host-specific gut microbiota composition. We here addressed whether peroral fecal microbiota transplantation (FMT) might be an antibiotics-independent option to lower even high gastrointestinal *C. jejuni* loads in the infected vertebrate host. To address this, secondary abiotic mice were generated by broad-spectrum antibiotic treatment and perorally infected with *C. jejuni* by gavage. One week later, mice were stably colonized with more than 10^9^ *C. jejuni* and subjected to peroral FMT from murine donors on three consecutive days. Two weeks post-intervention, gastrointestinal *C. jejuni* loads were up to 7.5 orders of magnitude lower following murine FMT versus mock challenge. Remarkably, FMT reversed *C. jejuni* induced colonic epithelial apoptosis, but enhanced proliferative and regenerative responses in the colon thereby counteracting pathogenic cell damage. Furthermore, FMT dampened both, innate and adaptive immune cell responses in the large intestines upon *C. jejuni* infection that were accompanied by less *C. jejuni*-induced colonic nitric oxide secretion. Our study provides strong evidence that novel probiotic formulations developed as alternative option to FMT in severe intestinal inflammatory morbidities including *Clostridoides difficile* infection might be effective to treat campylobacteriosis and lower pathogen loads in colonized vertebrates including farm animals.

## Introduction

*Campylobacter jejuni* constitute the most common agents of bacterial gastroenteritis in industrialized nations, causing more than 240,000 cases in the European Union per year^1^. According to the European Food Safety Authority (EFSA) and the European Center for Disease Prevention and Control (ECDC), campylobacteriosis represents the most reported zoonosis in the European Union, by far outnumbering salmonellosis, yersinosis and diseases caused by pathogenic variants of *Escherichia coli*^2^. The Gram-negative bacteria are transmitted to humans via contaminated food products including meat, milk and eggs from livestock animals, particularly poultry, or surface water. Up to 40% of fresh broiler and turkey meat have been tested positive for *Campylobacter* species recently^2^. Whereas *C. jejuni* is equipped with a multi-facetted arsenal of virulence factors, humans can be infected with an infectious dose of 500 to 800 bacterial cells only, regardless of their health conditions^3–5^. Following predominant colonization of the terminal ileum and colon, the pathogen invades colonic epithelial cells and induces mucosal pro-inflammatory immune responses leading to crypt abscesses and focal ulcerations^6–8^. After an incubation period of 2 to 5 days, infected patients present with a broad variety of symptoms ranging from only mild malaise to fever, abdominal cramps, myalgia, and watery or even bloody diarrhea^7–9^. In rare cases, however, post-infectious sequelae such as Guillain-Barré syndrome, Bickerstaff encephalitis, Miller Fisher syndrome, Reiter’s syndrome and chronic intestinal inflammatory morbidities might arise with a latency of weeks to months^10,11^. Whereas the majority of infections is self-limited and require symptomatic treatment such as fluid replacement only, particularly infected multi-morbid patients with immune-suppressive comorbidities are subjected to antibiotic treatment. This intervention, however, is paid by expense of the potential antibiotics-induced collateral damages leading to a compromised commensal gut microbiota composition subsequently facilitating (opportunistic) pathogenic colonization and infection of the gastrointestinal tract, for instance^12–15^ besides to unwanted effects to the vertebrate immune system^16^. It is thus utmost appreciable to search for antibiotics-independent approaches for the prevention and treatment of *C. jejuni* colonization and infection in farm animals and humans, respectively.

In this context, one needs to take into consideration that it is the host-specific composition of the commensal gut microbiota determining whether the vertebrate host is susceptible towards or resistant against *C. jejuni* infection. Conventionally colonized wildtype mice, for instance, are protected from stable *C. jejuni* colonization and infection even upon peroral infection with high pathogenic doses^17–19^. Following murine microbiota depletion and hence, abrogation of the physiological colonization resistance by broad-spectrum antibiotic treatment, however, secondary abiotic mice, but also secondary abiotic mice that had been reconstituted with a complex human as opposed to a murine gut microbiota following fecal microbiota transplantation (FMT) could be stably colonized with the pathogen at high loads, but did not develop overt pathogen-induced symptoms such as wasting or bloody diarrhea^17^.

Therapeutic application of FMT has been already reported since the fourth century during the Chinese Dong-jin dynasty^20^. Lately, FMT has experienced a renaissance as a promising treatment option of recurrent and refractory *Clostridoides difficile* toxin mediated pseudomembranous colitis in antibiotics-pretreated patients, of inflammatory bowel morbidities such as ulcerative colitis, irritable bowel syndrome and constipation, of obesity and diabetes and of chronic fatigue syndrome^21–25^.

Based upon these intriguing results in mice and men, we addressed in the present antibiotic-independent intervention/treatment study whether murine FMT in secondary abiotic mice that were harboring the pathogen in their gastrointestinal tract (GIT) at high loads could i.) sufficiently lower pathogenic burdens, ii.) alleviate gut epithelial cell damage and iii.) dampen intestinal pro-inflammatory immune responses upon *C. jejuni* infection. Our preclinical intervention study provides strong evidence that FMT from distinct donors might represent promising options for the prevention and/or treatment of *C. jejuni* colonization and/or infections in farm animals and/or humans, respectively.

## Results

### Gastrointestinal pathogen burdens following murine fecal microbiota transplantation in *C. jejuni* infected secondary abiotic mice

**Figure 1:**
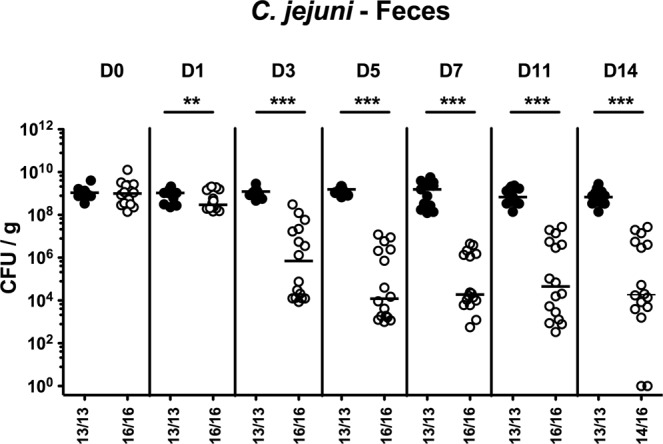
Intestinal pathogenic burdens over time following murine fecal microbiota transplantation of *C. jejuni* infected secondary abiotic mice. Secondary abiotic mice were infected with *C. jejuni* on days 0 and 1 by gavage. Starting a week later, infected mice were subjected to peroral fecal microbiota transplantation (FMT; Day (D) 0) from murine donors (open circles) on three consecutive days or received vehicle (mock; closed circles). Before and after FMT, *C. jejuni* loads were determined in fecal samples taken at indicated time points by culture and expressed as colony forming units per gram (CFU/g). Medians, numbers of culture-positive mice out of the total numbers of analyzed animals (in parentheses) and significance levels (p-values; **p < 0.01; ***p < 0.001) determined by the Mann-Whitney U test are given. Data were pooled from three independent experiments.

Secondary abiotic mice were perorally infected with 10^9^ viable *C. jejuni* strain 81–176 by gavage on days 0 and 1 and harbored median loads of approximately 10^9^ colony forming units (CFU) of the pathogen per g feces in both, the FMT and mock intervention cohorts a week later. On days 7, 8 and 9 post-infection (p.i.), mice were either subjected to murine FMT or received vehicle (D0, D1, D2). Cultural analyses of fecal samples revealed that as early as 24 hours after the latest murine FMT (i.e., 10 days post-infection), fecal *C. jejuni* loads were significantly lower in the FMT versus the mock cohort (p < 0.01; Fig. 1). Until the end of the observation period (i.e., two weeks after the initial FMT; 21 days post-infection), median intestinal pathogenic burdens had decreased by up to 5 log orders of magnitude following murine FMT (p < 0.001, Fig. 1; Supplementary Information), but remained stable in mock control mice (n.s.; Supplementary Information). Of note, 12.5% of mice from the FMT cohort, but none of the mock controls had completely cleared the pathogen from their intestines (Supplementary Information).

**Figure 2:**
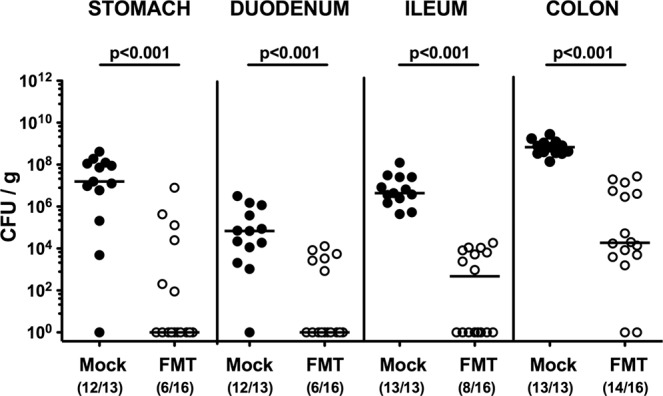
Gastrointestinal pathogenic loads following murine fecal microbiota transplantation of *C. jejuni* infected secondary abiotic mice. Secondary abiotic mice were infected with *C. jejuni* on days 0 and 1 by gavage. Starting a week later, infected mice were subjected to peroral fecal microbiota transplantation (FMT) from murine donors (open circles) on three consecutive days or received vehicle (mock; closed circles). On day 21 post-infection (i.e., 14 days post FMT), *C. jejuni* loads were determined in luminal samples taken from stomach, duodenum, ileum, and colon by culture and expressed as colony forming units per gram (CFU/g). Medians, numbers of pathogen-positive mice out of the total number of analyzed animals (in parentheses) and significance levels (p-values) determined by the Mann-Whitney U test are given. Data were pooled from three independent experiments.

**Figure 3:**
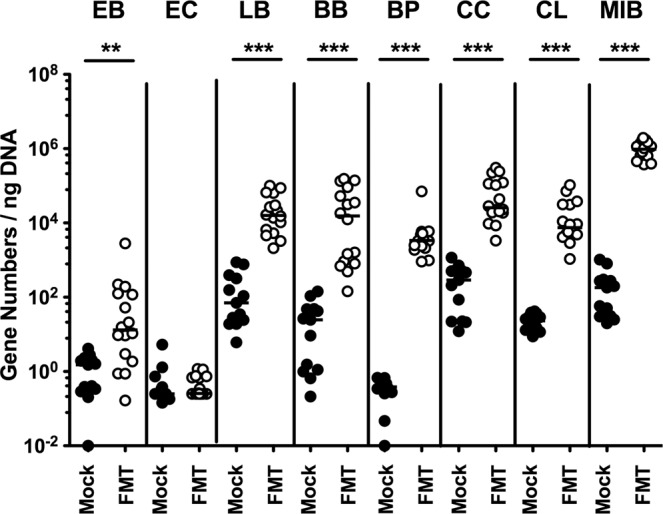
Fecal commensal microbiota composition following fecal microbiota transplantation in *C. jejuni* infected secondary abiotic mice. Secondary abiotic mice were infected with *C. jejuni* on days 0 and 1 by gavage. Starting a week later, infected mice were subjected to peroral fecal microbiota transplantation (FMT) from murine donors (open circles; n = 16) on three consecutive days or received vehicle (mock; closed circles; n = 13). On day 21 post-infection (i.e., 14 days post FMT), the fecal commensal microbiota composition was surveyed by culture-independent 16S rRNA methods quantitating the main bacterial groups and species including enterobacteria (EB), enterococci (EC), lactobacilli (LB), bifidobacteria (BB), *Bacteroides/Prevotella* (BP) species, *Clostridium coccoides* group (CC), *Clostridium leptum* group (CL) and *Mouse Intestinal Bacteroides* (MIB) and expressed as gene numbers per ng DNA. Medians and significance levels (p-values; **p < 0.01; ***p < 0.001) determined by the Mann-Whitney U test are given. Data were pooled from three independent experiments.

Upon necropsy (i.e., 21 days post-infection, 14 days post-FMT), we assessed *C. jejuni* loads alongside the GIT. Following murine FMT, *C. jejuni* counts were lower in luminal samples taken from stomach, duodenum, ileum, and colon as compared to mock control mice (p < 0.001; Fig. 2). Our culture-independent analyses further confirmed that the murine microbiota had sufficiently established in the large intestines of mice following FMT given that gene numbers of the main bacterial species and groups abundant in the murine gut microbiota including enterobacteria, enterococci, lactobacilli, bifidobacteria, *Bacteroides/Prevotella* species, *Clostridium coccoides* group, *Clostridium leptum* group, and *Mouse Intestinal Bacteroides* were comparable in the murine fecal donor transplants and in the gut microbiota following murine FMT (Fig. 3; Supplementary Information).

Thus, FMT could sufficiently lower gastrointestinal pathogenic loads in *C. jejuni* infected mice, even upon high-dose infection.

### Apoptotic and proliferating colonic epithelial cell responses following murine fecal microbiota transplantation in *C. jejuni* infected secondary abiotic mice

**Figure 4:**
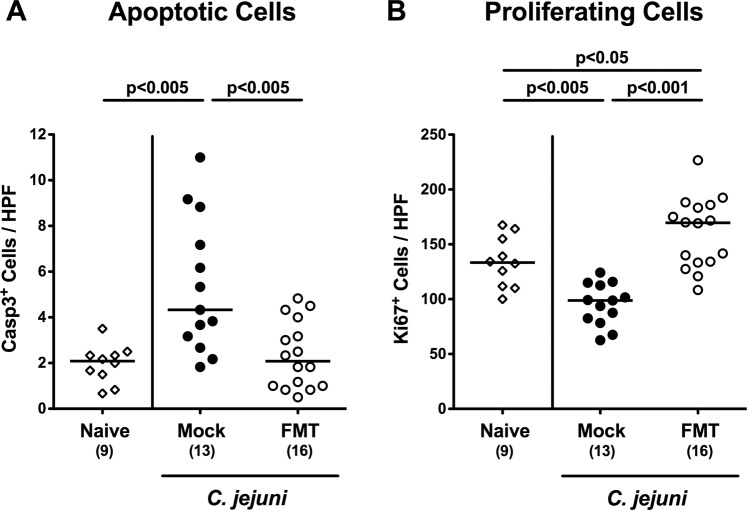
Apoptotic and proliferating colonic epithelial cell responses upon fecal microbiota transplantation of *C. jejuni* infected secondary abiotic mice. Secondary abiotic mice were infected with *C. jejuni* on days 0 and 1 by gavage. Starting a week later, infected mice were subjected to peroral fecal microbiota transplantation (FMT) from murine donors (open circles) on three consecutive days or received vehicle (mock; closed circles). On day 21 post-infection (i.e., 14 days post FMT), the average numbers of (**A**) apoptotic (caspase3, Casp3+) and (**B**) proliferating (Ki67+) colonic epithelial cells were determined from six high power fields (HPF, 400 × magnification) per mouse in immunohistochemically stained large intestinal paraffin sections. Naive secondary abiotic mice served as uninfected and untreated controls (open diamonds). Medians, numbers of analyzed animals (in parentheses) and significance levels (p-values) determined by the Mann-Whitney U test are given. Data were pooled from three independent experiments.

In order to address whether murine FMT resulted in less distinct *C. jejuni* induced intestinal cell damage, we quantitatively assessed cleaved caspase3+ apoptotic cells in colonic paraffin sections applying *in situ* immunohistochemistry. Whereas within 21 days *C. jejuni* had induced marked increases in apoptotic colonic epithelial cell numbers in mock control mice (p < 0.005; Fig. 4A; Supplementary Information), this was not the case following murine FMT (p < 0.005 vs mock; Fig. 4A; Supplementary Information). We further quantitated Ki67+ colonic epithelial cells indicative for cell proliferation and regeneration. Whereas Ki67+ colonic epithelial cells decreased upon *C. jejuni* infection of mock control animals (p < 0.005) proliferating cell numbers were even higher in infected mice of the FMT cohort as compared to uninfected and untreated (i.e., naive) mice (p < 0.05; Fig. 4B; Supplementary Information). Thus, murine FMT prevented from *C. jejuni* induced colonic epithelial apoptosis and enhanced proliferative and regenerative cell responses in the large intestines counteracting pathogenic cell damage.

### Large intestinal inflammatory immune responses upon murine fecal microbiota transplantation of *C. jejuni* infected mice

**Figure 5:**
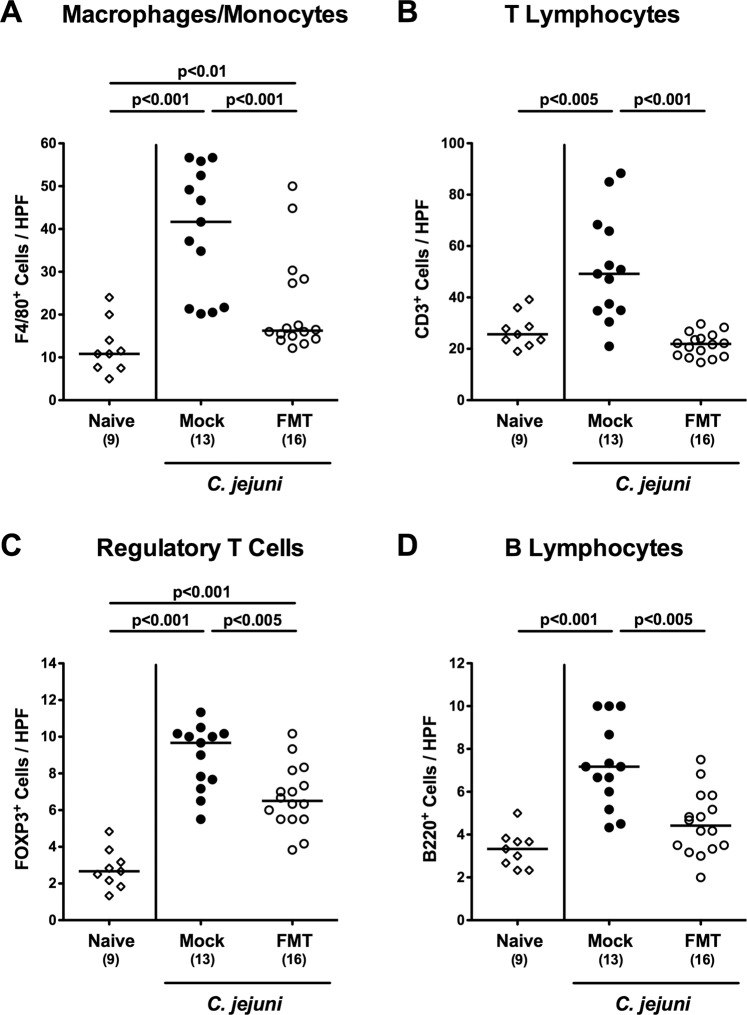
Colonic immune cells responses upon fecal microbiota transplantation of *C. jejuni* infected secondary abiotic mice. Secondary abiotic mice were infected with *C. jejuni* on days 0 and 1 by gavage. Starting a week later, infected mice were subjected to peroral fecal microbiota transplantation (FMT) from murine donors (open circles) on three consecutive days or received vehicle (mock; closed circles). On day 21 post-infection (i.e., 14 days post FMT), the average numbers of **(A)** macrophages and monocytes (F4/80+), **(B)** T lymphocytes (CD3+), **(C)** regulatory T cells (FOXP3+), and **(D)** B lymphocytes (B220+) in the large intestinal mucosa and lamina propria were determined from six high power fields (HPF, 400 x magnification) per mouse in immunohistochemically stained large intestinal paraffin sections. Naive secondary abiotic mice served as uninfected and untreated controls (open diamonds). Medians, numbers of analyzed animals (in parentheses) and significance levels (p-values) determined by the Mann-Whitney U test are given. Data were pooled from three independent experiments.

We next surveyed innate and adaptive immune cell responses in the large intestines of *C. jejuni* infected mice following FMT applying quantitative *in situ* immunohistochemistry. At day 21 p.i., mice displayed multi-fold increased numbers of innate immune cell subsets such as F4/80+ macrophages and monocytes in their colonic mucosa and lamina propria (p < 0.01–0.001 vs naive), but far less distinctly in mice of the FMT cohort (p < 0.001 vs mock; Fig. 5A; Supplementary Information). Furthermore, adaptive immune cell populations such as CD3+ lymphocytes and B220+ B lymphocytes had increased within 21 days post-infection of mock (p < 0.005 and p < 0.001 vs naive, respectively), but not FMT treated mice (n.s. vs naive; p < 0.005–0.001 vs mock; Fig. 5B,D; Supplementary Information). As for F4/80+ cells, *C. jejuni* induced increases in FOXP3+ regulatory T cell numbers were less pronounced in the colonic mucosa and lamina propria following FMT versus mock challenge at day 21 p.i. (p < 0.005; Fig. 5C; Supplementary Information).

**Figure 6:**
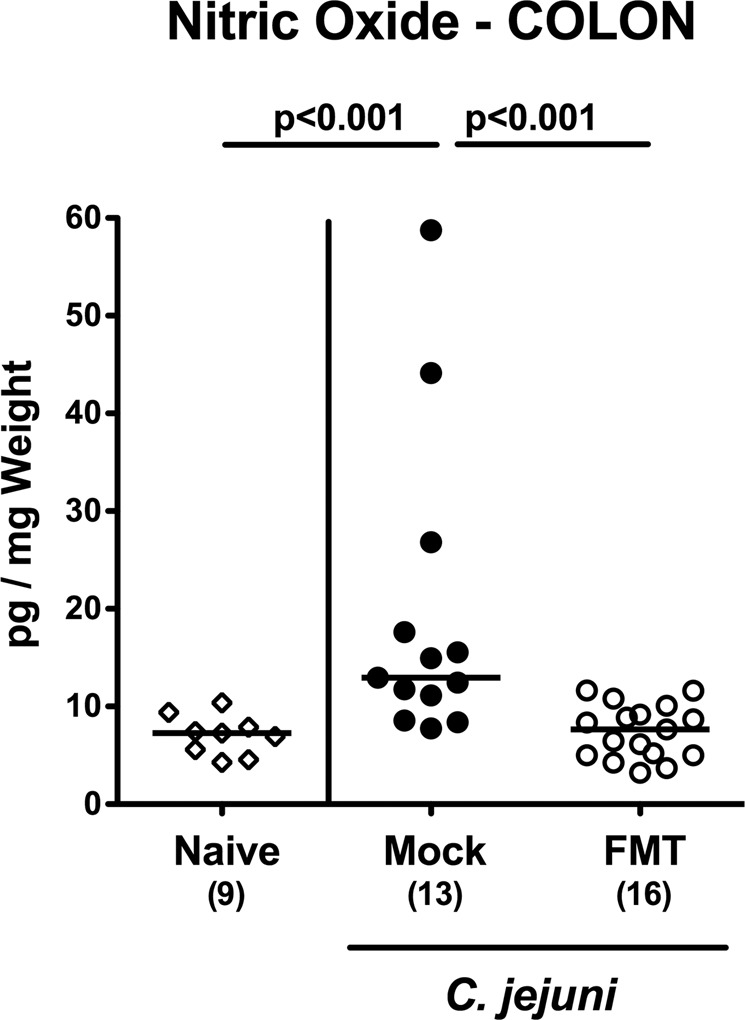
Colonic nitric oxide secretion upon fecal microbiota transplantation of *C. jejuni* infected secondary abiotic mice. Secondary abiotic mice were infected with *C. jejuni* on days 0 and 1 by gavage. Starting a week later, infected mice were subjected to peroral fecal microbiota transplantation (FMT) from murine donors (open circles) on three consecutive days or received vehicle (mock; closed circles). On day 21 post-infection (i.e., 14 days post FMT), nitric oxide concentrations were measured in supernatants of colonic *ex vivo* biopsies. Naive secondary abiotic mice served as uninfected and untreated controls (open diamonds). Medians, numbers of analyzed animals (in parentheses) and significance levels (p-values) determined by the Mann-Whitney U test are given. Data were pooled from three independent experiments.

We further addressed whether less distinct colonic immune cell responses upon FMT of *C. jejuni* infected mice were accompanied with less intestinal pro-inflammatory mediator secretion. In fact, nitric oxide concentrations were elevated in colonic *ex vivo* biopsies taken from mock control animals (p < 0.001 vs naive), but not from mice of the FMT cohort at day 21 p.i. (p < 0.001 vs mock; Fig. 6).

Thus, murine FMT could dampen both, innate and adaptive immune responses in the large intestines upon *C. jejuni* infection that was accompanied by less *C. jejuni* induced colonic nitric oxide secretion. Furthermore, murine FMT promoted Ki67 positive cell regenerative properties also in the colon of *C. jejuni* infected mice.

## Discussion

The physiological colonization resistance protecting the vertebrate host from unwanted pathogenic colonization and infection provides us promising opportunities to identify distinct molecules and gut bacterial populations as potential novel options for the prevention and treatment of campylobacterisis and its post-infectious sequelae. We are currently, however, still at the beginning of understanding the underlying mechanisms. In our actual antibiotics-independent intervention study applying a murine high dose *C. jejuni* infection model, we were able to show that murine FMT treatment of secondary abiotic mice harboring very high pathogenic loads of approximately 10^9^ viable bacteria per g intestinal luminal sample lowered gastrointestinal *C. jejuni* loads up to 7.5 orders of magnitude within two weeks post-intervention. This pronounced pathogen-lowering effect is even more intriguing when considering the low infectious doses of a few hundred bacterial cells needed to induce campylobacteriosis in humans^5^. Besides the substantial pathogen-lowering effect, murine FMT did also affect the murine *C. jejuni* carrier rates given that 12.5% of high-dose infected mice had even completely expelled the pathogen from their GIT. Remarkably, FMT could effectively reverse *C. jejuni* induced colonic epithelial apoptosis, and enhance proliferative and regenerative cell responses in the large intestines thereby counteracting pathogenic cell damage. Furthermore, FMT dampened both, innate and adaptive immune cell responses in the large intestines upon *C. jejuni* infection that was accompanied by less secretion of nitric oxide exerting oxidative stress to the intestinal epithelia^26,27^. In support, we were able to show very recently that murine FMT could decrease intestinal loads of the Gram-negative opportunistic pathogen *Pseudomonas aeruginosa* in murine carriers^28^. The immune cell function restoring properties of murine FMT in secondary abiotic mice was convincingly shown by Ekmekciu and coworkers previously. Whereas gut microbiota depletion by broad-spectrum antibiotic treatment resulted in profound changes of the immune cell repertoire in local intestinal and systemic compartments, reintroduction of the complex murine gut microbiota by peroral FMT could reverse the collateral damages of antibiotic treatment^16^.

A key question is whether the entire complex gut microbiota, defined bacterial species alone or in combination, and/or distinct molecules shaping a (for the pathogen) hostile intraluminal milieu within the GIT are needed to expel the pathogen from already taken ecological niches. Our recent study revealed that application of a single *Lactobacillus johnsonii* strain that had been isolated from a healthy conventional C57BL/6j mouse resulted in ameliorated intestinal, extra-intestinal and even systemic immune responses upon high-dose *C. jejuni* infection of secondary abiotic mice in a time-of-treatment dependent fashion^29^, but without having a pathogen-lowering effect. Ekmekciu *et al*. further addressed the capabilities of defined Gram-positive and Gram-negative intestinal commensals such as *Lactobacillus johnsonii* and *Escherichia coli*, respectively, to restore cellular immune functions as compared to complex microbiota in mice that had been subjected to broad-spectrum antibiotic treatment with subsequent immune-suppressive sequelae^30^. In fact, FMT appeared to be most potent to reverse antibiotics-induced immune dysfunction, whereas respective commensal species could effectively restore individual functions of both, intestinal and systemic immunity. Furthermore, recolonization of secondary abiotic mice with the probiotic formulation VSL#3 consisting of eight bacterial strains (i.e., four different *Lactobacillus* species, three *Bifidobacterium* species and *Streptococcus thermophilus*), has been shown to reverse antibiotics-induced immune cellular dysfunction and to provide anti-inflammatory responses in intestinal mucosal and systemic compartments^31^. Notably, probiotic VSL#3 treatment of *C. jejuni* infected secondary abiotic mice could alleviate intestinal and extra-intestinal including systemic sequelae of infection^32^.

Nevertheless, it appears to be rather a search for the needle in the hay-stack when trying to identify effective pathogen-lowering and immune-modulatory (probiotic) bacterial strains. It is rather more likely, that literally, the orchestrated interplay of commensals with the immune system as the conductor constitutes the most promising approach to tackle intestinal pathogenic colonization/infection of vertebrates.

From disease-preventive strategies of human campylobacteriosis, however, it is of even more importance to lower the intestinal *C. jejuni* loads in poultry and other livestock animals for subsequent risk reduction of pathogenic transmission to human via the food chain. In support, both, prophylactic and therapeutic application of a commercial bacterial formulation (Aviguard®) has been successfully shown to compete with intestinal colonization and expansion, respectively, of chicken with unwanted bacteria such as *Clostridium perfringens*^33,34^ and *Salmonella typhimurium*^35^ additionally resulting in less distinct intestinal inflammatory sequelae of colonization/infection. We are currently evaluating potential intestinal pathogenic burden lowering and anti-inflammatory effects of prophylactic and therapeutic application in high-dose *C. jejuni* infected mice.

### In conclusion

Our intervention study provides strong evidence that active modulation of the microbiota composition by murine FMT might be considered an effective measure to lower even high intestinal *C. jejuni* loads and dampen pathogen-induced inflammatory immune responses in colonized/infected vertebrates including farm animals. Subsequent application-oriented efforts to develop pharmaceutical probiotic formulations that might even replace FMT in clinical practice^36^ would provide novel options for a safe, easy-to-handle and (hopefully) cost-efficient treatment of *C. jejuni* infections in humans. From the prophylactic perspective, efficient reduction of *C. jejuni* colonization in farm animals would minimize the risk of pathogenic transmission via the food chain, of human infection and of post-infectious sequelae.

## Materials and Methods

### Ethical statement

Mouse experiments were conducted in accordance with the European Guidelines for animal welfare (2010/63/EU) after approval by the commission for animal experiments headed by the “Landesamt für Gesundheit und Soziales” (LaGeSo, Berlin, registration numbers G0097/12 and G0039/15. One a day clinical conditions of mice were monitored.

### Generation of secondary abiotic mice

Conventional female and male C57BL/6j mice were bred and reared under specific pathogen free (SPF) conditions in the same unit of the Forschungseinrichtungen für Experimentelle Medizin (FEM, Charité - University Medicine Berlin). In order to counteract physiological colonization resistance, secondary abiotic mice with a depleted gut microbiota were generated as described previously^17,26^. In brief, upon weaning 3-week old mice were placed into autoclaved cages and subjected to broad-spectrum antibiotic treatment for 8 weeks (ampicillin plus sulbactam (1 g/L; Ratiopharm, Germany), vancomycin (500 mg/L; Cell Pharm, Germany), ciprofloxacin (200 mg/L; Bayer Vital, Germany), imipenem (250 mg/L; MSD, Germany) and metronidazole (1 g/L; Fresenius, Germany)) added to autoclaved tap water. To control the intestinal colonization status and absence of cultivable bacteria in the murine intestinal tract, individual fecal samples were derived once a week as well as immediately before the first *C. jejuni* infection and incubated in thioglycolate broths (Oxoid) for one week at 37 °C. Bacterial growth was monitored daily by turbidity assessment. Aliquots from turbid broths as well as from broths remaining clear after one week of incubation were cultivated on solid media under aerobic, microaerobic and obligate anaerobic conditions and grown bacteria identified microscopically and biochemically as described^26^. Virtual absence of fastidious and uncultivable bacteria in intestinal tract of secondary abiotic mice has been confirmed in our previous study by quantitative 16S rRNA based PCR analyses showing that bacterial gene numbers in fecal samples following quintuple antibiotic treatment were comparable to those detected in autoclaved food pellets^16^. Three days before start of the infection experiments the antibiotic cocktail was replaced by autoclaved tap water (*ad libitum*).

### *C. jejuni* infection and colonization properties

At days 0 and 1, sex and age matched secondary abiotic mice were perorally infected with 10^9^ colony forming units (CFU) of the *C. jejuni* 81–176 strain by gavage (in a total volume of 0.3 mL phosphate buffered saline (PBS, Gibco, Life Technologies, UK)). Animals remained in a sterile environment (with autoclaved food and drinking water or sterile antibiotic cocktail) and handled under strictly aseptic conditions to avoid contaminations.

*C. jejuni* loads were quantitatively assessed in fecal samples over time post-infection and upon necropsy (i.e., at day 21 p.i.) in luminal samples derived from the stomach, duodenum, ileum, and colon by culture as reported earlier^17,37^. The detection limit of viable pathogens was 100 CFU per g.

### Fecal microbiota transplantation

On days 7, 8 and 9 p.i., mice were subjected to peroral FMT from murine donors on three consecutive days. Fresh murine fecal samples were collected from 10 age and sex matched SPF control mice, pooled, dissolved in 10 mL sterile PBS and the supernatant taken as murine donor suspension. Aliquots from each murine fecal donor solutions were collected for quantitative molecular analyses of main intestinal bacterial communities as reported previously^17,26,38^ (Supplementary Information).

### Molecular analysis of gut microbiota composition

DNA was extracted from fecal and colonic luminal samples as well as from fecal docor suspensions as described previously^26,39^. In brief, DNA was quantified by using Quant-iT PicoGreen reagent (Invitrogen, UK) and adjusted to 1 ng per µL. Then, the main bacterial species and groups abundant in the murine gut microbiota including enterobacteria, enterococci, lactobacilli, bifidobacteria, *Bacteroides/Prevotella* spp., *Clostridium coccoides* group, *Clostridium leptum* group, and *Mouse Intestinal Bacteroides* were assessed by quantitative real-time polymerase chain reaction (qRT-PCR) with species-, genera- or group-specific 16S rRNA gene primers (Tib MolBiol, Germany) as stated elsewhere^17,38,40^ and expressed as numbers of 16S rRNA gene copies per ng DNA.

### Sampling procedures

Mice were sacrificed at day 21 p.i. (i.e., 14 days post-FMT) by isofluran inhalation (Abbott, Germany). Luminal samples were derived from distinct parts of the GIT (i.e., from stomach, duodenum ileum, and colon) and *ex vivo* biopsies from the large intestines were taken under sterile conditions. From each mouse colonic samples were collected in parallel for microbiological, immunohistopathological and immunological analyses.

### Immunohistochemistry

Colonic *ex vivo* biopsies were immediately fixed in 5% formalin, embedded in paraffin and subjected to *in situ* immunohistochemical analyses as stated elsewhere^38,41–43^. In brief, apoptotic epithelial cells, proliferating epithelial cells, macrophages/monocytes, T lymphocytes, regulatory T cells, and B lymphocytes were quantitated in paraffin sections (5 μm) that had been stained with primary antibodies against cleaved caspase 3 (Asp175, Cell Signaling, Beverly, MA, USA, 1:200), Ki67 (TEC3, Dako, Denmark, 1:100), F4/80 (# 14–4801, clone BM8, eBioscience, San Diego, CA, USA, 1:50), CD3 (#N1580, Dako, 1:10), FOXP3 (FJK-16s, eBioscience, 1:100), and B220 (No. 14-0452-81, eBioscience; 1:200), respectively. Positively stained cells were counted by a blinded independent investigator (light microscopy, magnification 100 x and 400 x), and the average number of respective positively stained cells for each mouse determined within at least six high power fields (HPF, 0.287 mm^2^, 400 x magnification).

### Nitric oxide measurement

Colonic *ex vivo* biopsies were cut longitudinally, washed in PBS, and strips of approximately 1 cm^2^ tissues were placed in 24-flat-bottom well culture plates (Nunc, Germany) containing 500 μL serum-free RPMI 1640 medium (Gibco, life technologies, UK) supplemented with penicillin (100 U/mL) and streptomycin (100 µg/mL; PAA Laboratories, Germany). After 18 h at 37 °C, culture supernatants were subjected to nitric oxide concentration measurements applying the Griess reaction as stated elsewhere^26,27^.

### Statistical analysis

Medians and levels of significance were determined using Mann-Whitney test (GraphPad Prism v7, USA) as indicated. Two-sided probability (p) values ≤ 0.05 were considered significant. Experiments were reproduced twice.

## Supplementary information

Supplementary Information
