# Supplementary material for: Murine fecal microbiota transplantation lowers gastrointestinal pathogen loads and dampens pro-inflammatory immune responses in Campylobacter jejuni infected secondary abiotic mice
Source: Sci Rep. 2019 Dec 24;9:19797. doi: 10.1038/s41598-019-56442-7 (PMC6930309; doi:10.1038/s41598-019-56442-7)
Supplement: Supplementary file 1 — Supplementary Information [file 41598_2019_56442_MOESM1_ESM.pdf]

Murine fecal microbiota transplantation lowers gastrointestinal pathogen loads and dampens pro-inflammatory immune responses in *Campylobacter jejuni* infected secondary abiotic mice

Markus M. Heimesaat , Katharina Mrazek, and Stefan Bereswill

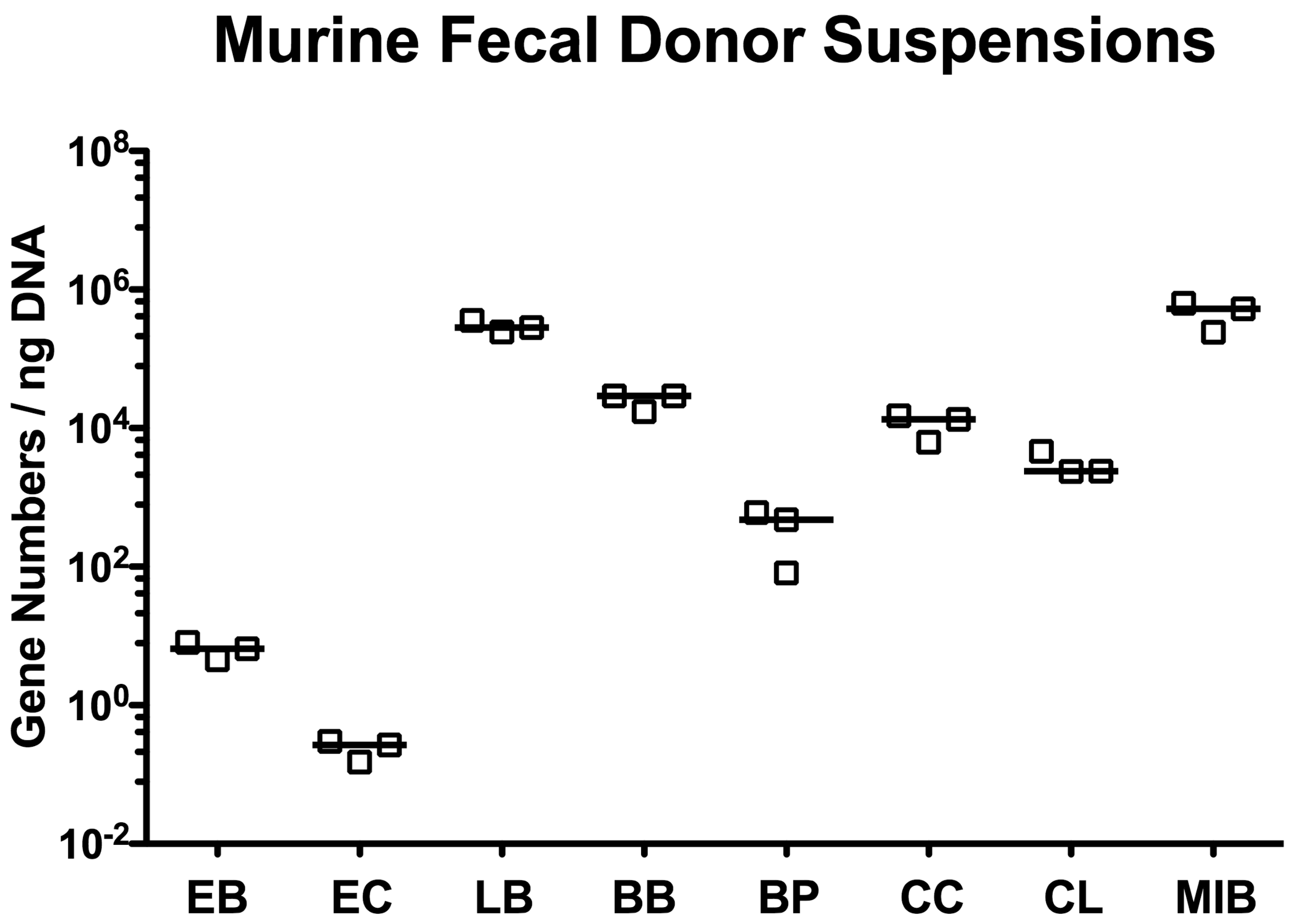

Microbiota composition of murine donor suspensions for fecal microbiota transplantation. Immediately before fecal microbiota transplantation the commensal microbiota composition of murine donor suspensions (n=3) was surveyed by culture-independent 16S rRNA methods quantitating main abundant bacterial groups including enterobacteria (EB), enterococci (EC), lactobacilli (LB), bifidobacteria (BB), *Bacteroides/Prevotella* species (BP), *Clostridium coccoides* group (CC), *Clostridium leptum* group (CL) and *Mouse Intestinal Bacteroides* (MIB). Bacterial loads are expressed as gene numbers per ng DNA und median indicated. Data are shown for one representative out of three independent experiments.

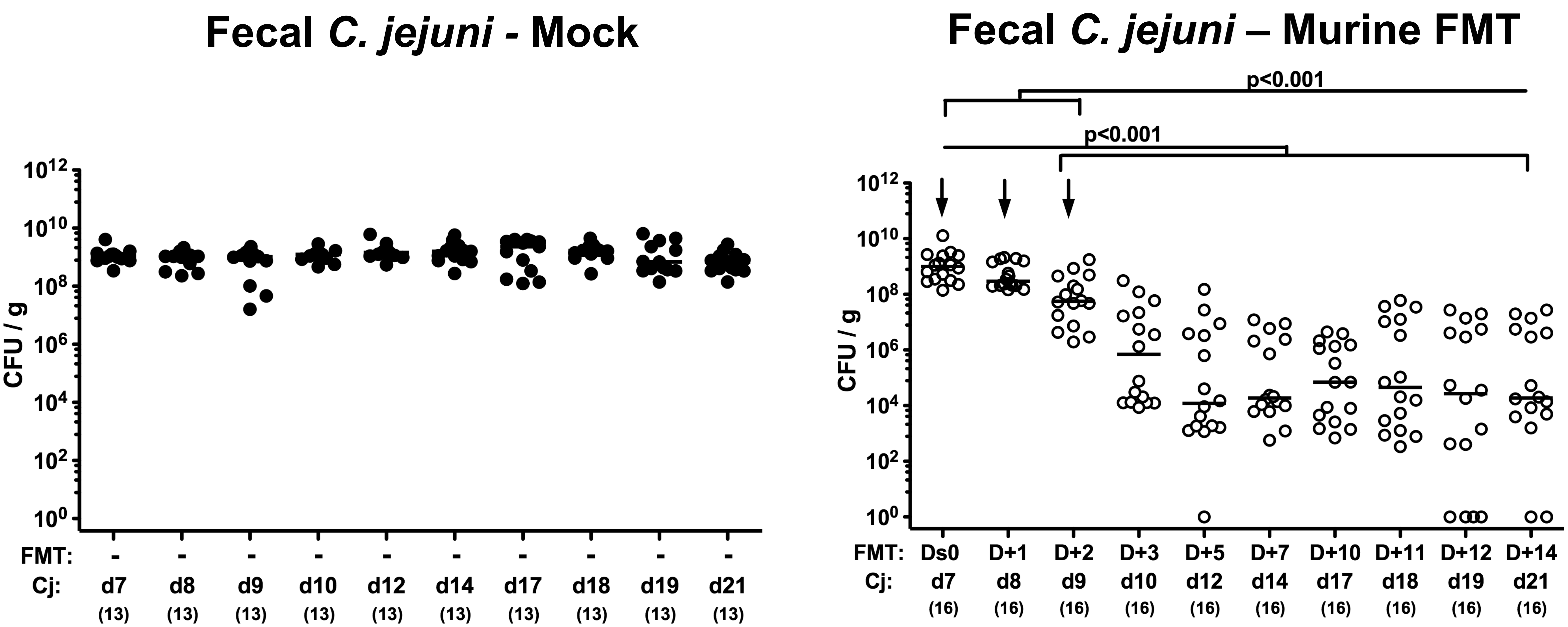

Kinetic survey of fecal *C. jejuni* shedding following murine fecal microbiota transplantation of *C. jejuni* infected secondary abiotic mice. Secondary abiotic mice were infected with *C. jejuni* (Cj) on day (d) 0 and d1 by gavage. Starting a week later, infected mice were subjected to peroral fecal microbiota transplantation (FMT) from murine donors (open circles) on three consecutive days or received vehicle (mock; closed circles) as indicated by arrows (Day (D) 0, D+1, D+2). Immediately before and after FMT, fecal samples were taken at defined time points to assess intestinal pathogenic loads by culture (expressed as colony forming units per gram, CFU / g). Medians, significance levels (p-values) determined by the Mann-Whitney U test and the total number of animals (in parentheses) are given. Data were pooled from three independent experiments.

Representative photomicrographs illustrating apoptotic and proliferating epithelial as well as immune cells responses in large intestines upon murine fecal microbiota transplantation in *C. jejuni* infected secondary abiotic mice. Secondary abiotic mice were infected with *C. jejuni* on days 0 and 1 by gavage. Starting a week later, infected mice were subjected to peroral fecal microbiota transplantation (FMT) from murine donors on three consecutive days or received vehicle (mock). Naive secondary abiotic mice served as uninfected and untreated controls. Photomicrographs reepresentative for three independent experiments illustrate the average numbers of **(A)** apoptotic epithelial cells (Casp3+), **(B)** proliferating epithelial cells (Ki67+), **(C)** macrophages and monocytes (F4/80+), **(D)** T lymphocytes (CD3+), **(E)** regulatory T cells (FOXP3+), and **(F)** B lymphocytes (B220+) in at least six high power fields (HPF) as quantitatively assessed in colonic paraffin sections applying *in situ* immunohistochemistry at day 21 post-infection (i.e., 14 days post FMT; 100 x magnification, scale bar 100 µm).

A Apoptotic Cells (COLON)

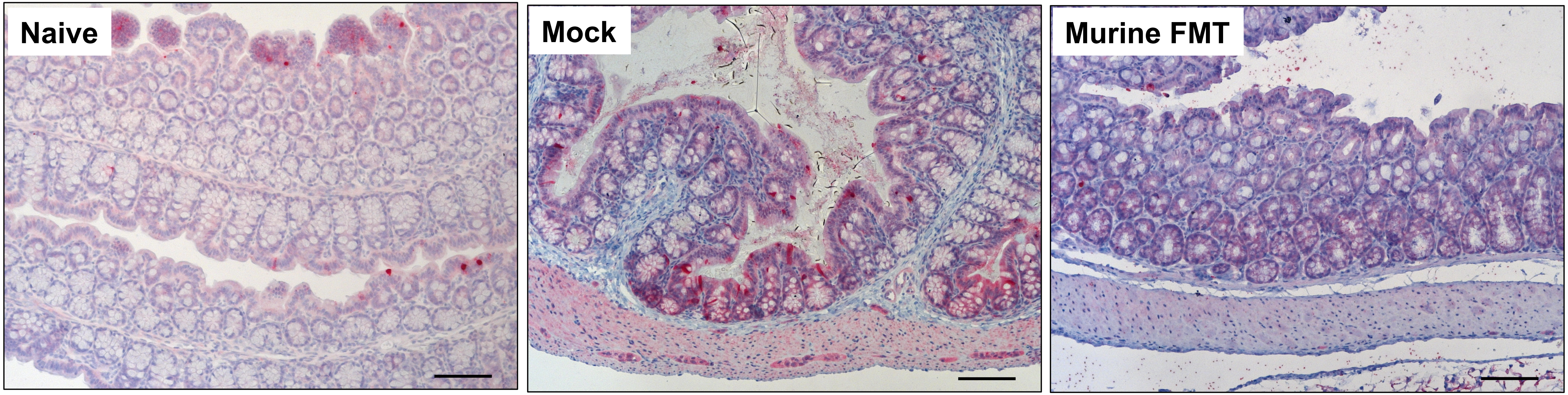

(100 x magnification, scale bar 100 µm)

B

Proliferating Cells (COLON)

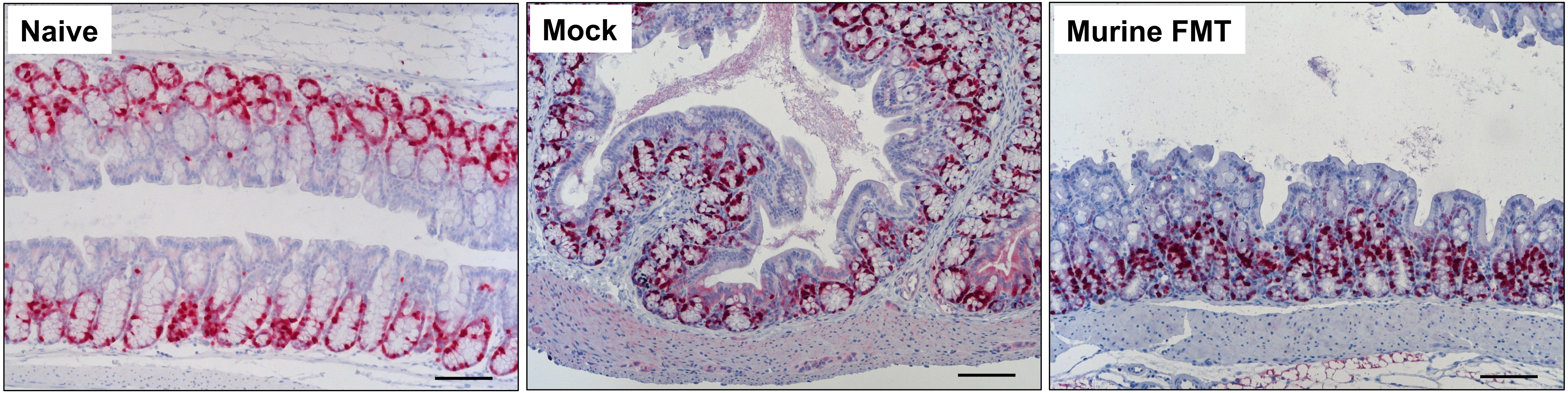

(100 x magnification, scale bar 100 μm)

C

Macrophages / Monocytes (COLON)

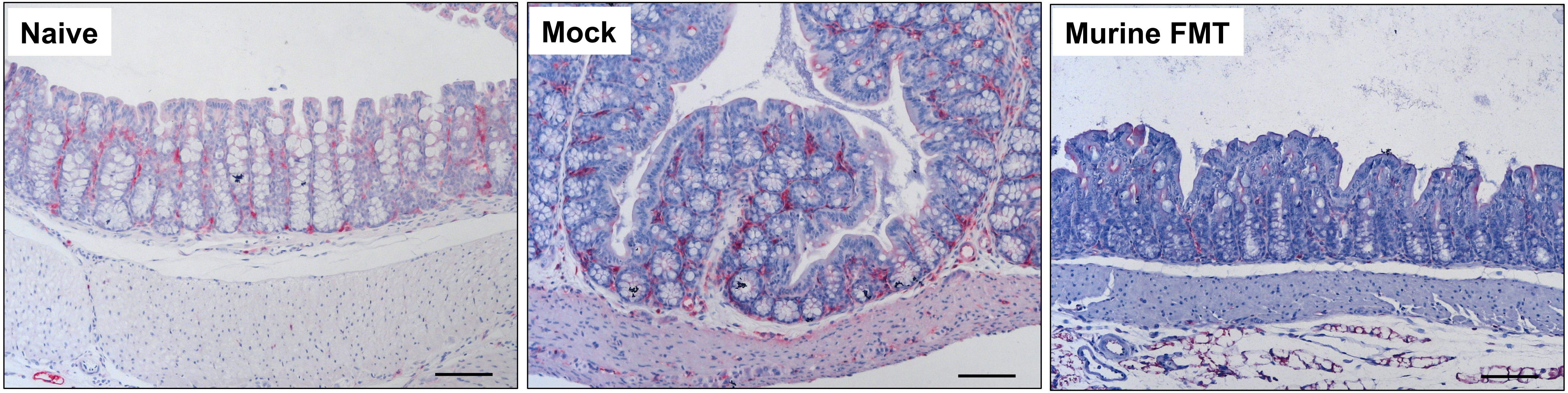

(100 x magnification, scale bar 100 µm)

D T Lymphocytes (COLON)

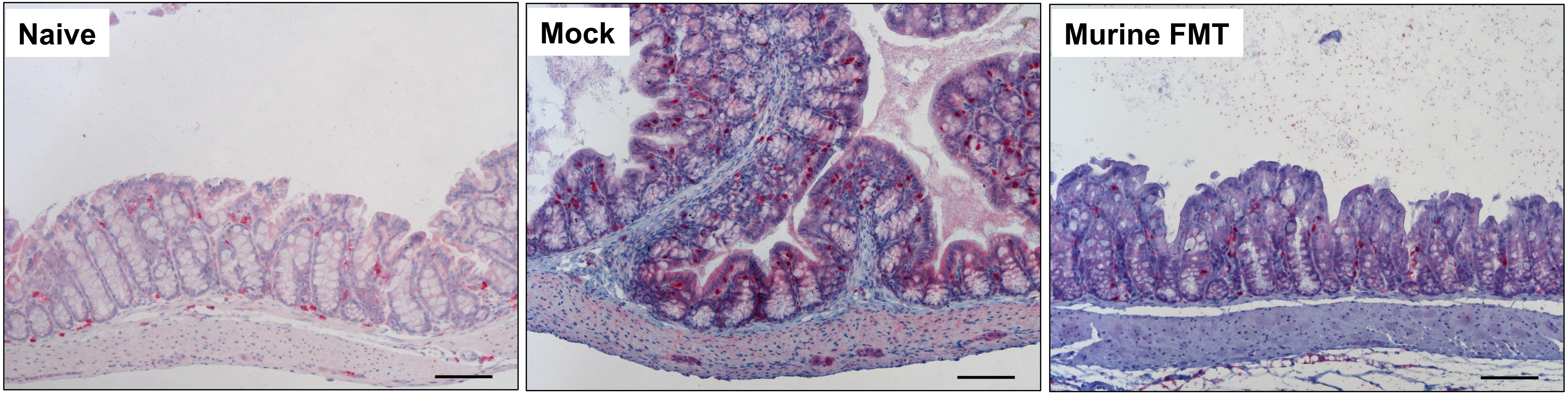

(100 x magnification, scale bar 100 µm)

E

Regulatory T Cells (COLON)

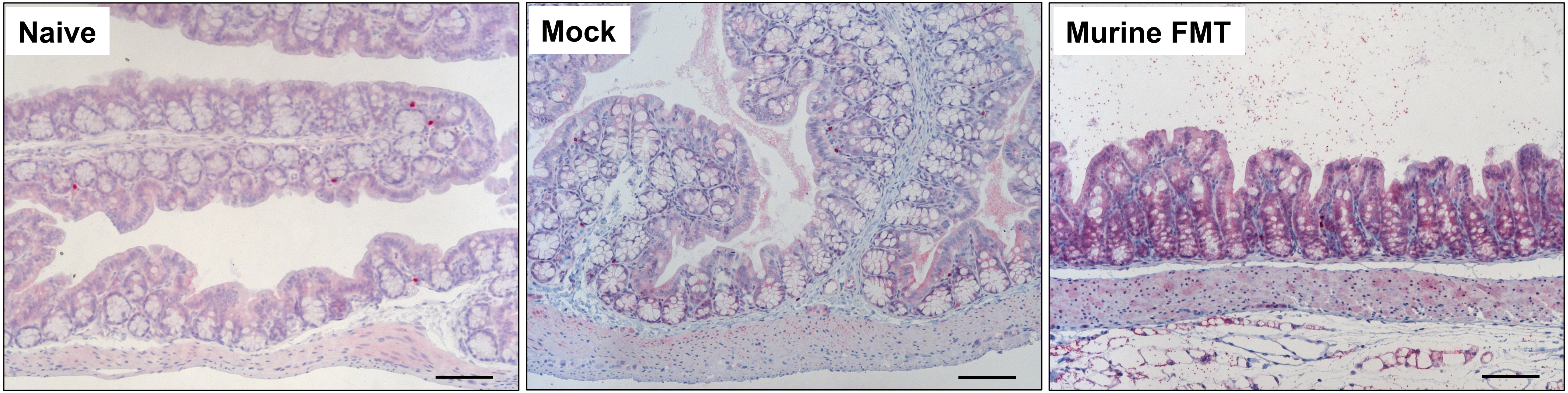

(100 x magnification, scale bar 100 μm)

F B Lymphocytes (COLON)

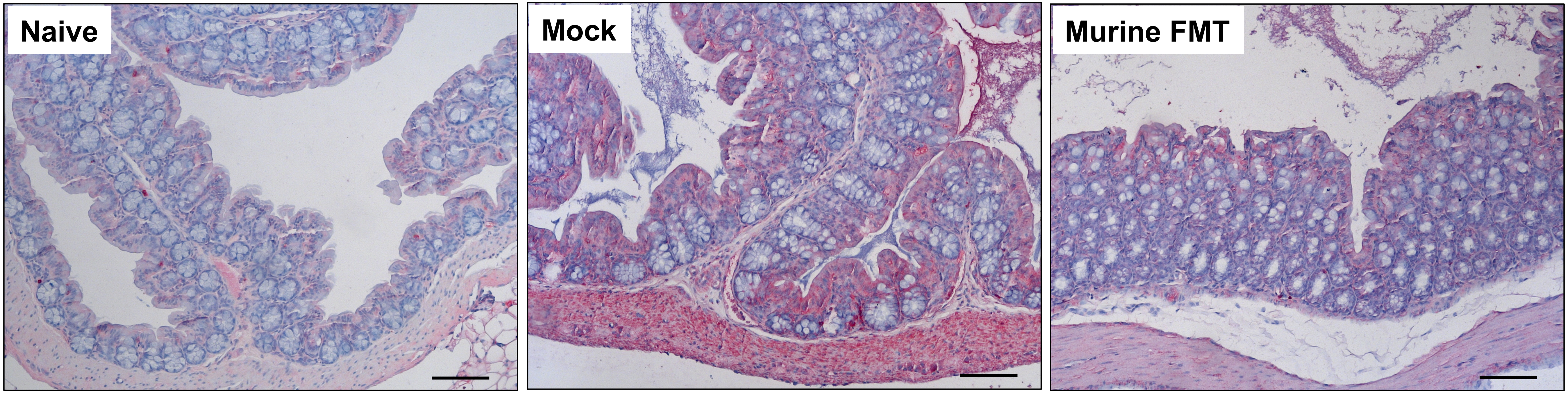

(100 x magnification, scale bar 100 µm)
